# Lidocaine as an element of multimodal analgesic therapy in major spine surgical procedures in children: a prospective, randomized, double-blind study

**DOI:** 10.1007/s43440-020-00100-7

**Published:** 2020-04-15

**Authors:** Ilona Batko, Barbara Kościelniak-Merak, Przemysław J. Tomasik, Krzysztof Kobylarz, Jerzy Wordliczek

**Affiliations:** 1 Department of Anesthesiology and Intensive Care, University Children’s Hospital, 265 Wielicka St, 30-663 Cracow, Poland; 2 Department of Clinical Biochemistry, University Children’s Hospital, Jagiellonian University Medical College, Cracow, Poland; 3 Department of Anesthesiology and Intensive Care, Jagiellonian University Medical College, Cracow, Poland; 4 Department of Interdisciplinary Intensive Care, Jagiellonian University Medical College, Cracow, Poland

**Keywords:** Lidocaine intravenous, Multimodal analgesia, Pediatric anesthesia, Postoperative pain, Spinal surgery

## Abstract

**Background:**

Introducing the principles of multimodal analgesic therapy is necessary to provide appropriate comfort for the patient after surgery. The main objective of the study was evaluating the influence of perioperative intravenous (i.v.) lidocaine infusion on postoperative morphine requirements during the first 48 h postoperatively in children undergoing major spine surgery.

**Materials and methods:**

Prospective, randomized, double-blind study: 41 children, qualified to multilevel spine surgery, were randomly divided into two treatment groups: lidocaine and placebo (control). The lidocaine group received lidocaine as a bolus of 1.5 mg/kg over 30 minutes, followed by a continuous infusion at 1 mg/kg/h to 6 hours after surgery. The protocol of perioperative management was identical for all patients. Measurements: morphine demand, intensity of postoperative pain (the Numerical Rating Scale), oral feeding initiation time, first attempts at assuming erect position, postoperative quality of life (the Acute Short-form /SF-12/ health survey).

**Results:**

Patient data did not differ demographically. Compared to the control group, lidocaine treatment reduced the demand for morphine during the first 24h [95% CI 0.13 (0.11-0.28) mg/kg,* p* = 0.0122], 48h [95% CI 0.46 (0.22-0.52) mg/kg,* p* = 0.0299] after surgery and entire hospitalization [95% CI 0.58 (0.19-0.78) mg/kg,* p* = 0.04]; postoperative pain intensity; nutritional withdrawal period [introduction of liquid diet (*p* = 0.024) and solid diet (*p* = 0.012)], and accelerated the adoption of an upright position [sitting (*p* = 0.048); walking (*p* = 0.049)]. The SF-12 generic health survey did not differ between groups before operation, 2 months and 4 years after surgery.

**Conclusions:**

Perioperative lidocaine administration, as a part of the applied analgesic therapy regimen, may decrease postoperative opioid demand and accelerates convalescence of children undergoing major surgery.

## Introduction

An extensive spinal procedure represents a major trauma. Inflammatory mediators released from damaged tissues initiate cascade reactions leading to a systemic inflammatory response syndrome, organ dysfunction and pain [1–3]. Multimodal analgesic therapy assumes that effective control of postoperative pain is achieved using a number of different analgesics and routes of administration, so that they act synergistically. This method uses the possibility of multidirectional inhibition of nociception process and enables continuous modulation of pain information flow. The goal of multimodal anesthesia is to improve pain relief, reduce opioid administration and opioid-related adverse effects, limit inflammatory reactions and prevent chronic postoperative pain development [4]. The techniques of regional blockades are difficult to perform and associated with an increased risk of complications in patients qualified to major spinal operations [5]. Alternate to supplement general anesthesia, adjuvant medications with analgesic, anti-inflammatory and antihyperalgesic properties are used. They are easy to apply and associated with a low percentage of adverse effects. To-date, these effects in spine surgery have been observed for combined therapy with gabapentin, pregabalin, ketamine, magnesium, dexamethasone and clonidine [2, 6–8]. Recently, it has been reported that lidocaine also beneficially supplements therapy aiming at improving patient comfort in the postoperative period [9–17]. However, there are no studies evaluating the intravenous (i.v) lidocaine as an element of analgesic multimodal therapy in spine surgery in children.

### Objective

The purpose of current study was to hypothesize that a perioperative lidocaine infusion would reduce opioid requirements during the first 48 h postoperative in pediatric patients undergoing major spine operations.

## Materials and methods

The protocol of the study was approved by the Jagiellonian University Bioethical Committee [No. 122.6120.89.2015]. All procedures performed in studies involving human participants were in accordance with the ethical standards of the institutional and national research committee and with the 1964 Helsinki Declaration and its later amendments or comparable ethical standards. All the parents or legal guardians of the patients as well patients over 16 years provided written informed consent prior to the inclusion in the study.

### Participants

This project was conducted at the University Pediatric Hospital in Cracow that performs 60–90 major spine operations annually. All children qualified to multilevel spinal surgery from May 2015 to June 2016 were assessed for study eligibility. The inclusion criteria were: age below 18 years, major spine surgery and the ASA (the American Society of Anesthesiologists physical status) I, II. The exclusion criteria were: liver and renal impairment, epilepsy, arrhythmia, long QT syndrome, allergy to lidocaine, obesity (body mass index > 30), chronic opioid therapy, history of organ transplantation and planned long-term postoperative mechanical ventilation.

### Randomization

The study we performed was randomized, double-blind, placebo-controlled. After qualification for the study, patients were randomly assigned to the lidocaine or the control group using a computer-generated random table and an allocation ratio of 1:1. The randomization sequence was generated by a hospital pharmacist who was not involved in the study. Before the surgery, the hospital pharmacist prepared a coded syringe which contained a blinded fluid (BF): lidocaine 20 mg/ml (Lignocainum hydrochloricum WZF 2%: Polfa S.A. Warsaw, Poland) or placebo -multi-electrolyte fluid (Fresenius Kabi, Warsaw, Poland). BF was administered intravenously as a half hour bolus before skin incision 0.075 ml/kg/30 min, and then continued intraoperatively for up to 6 h after surgery at a flow of 0.05 ml/kg/h. The medical personnel responsible for the perioperative patient care (anesthesiologists, surgeons, nurses) and the patient himself were blind to what BF was. The study coordinator supervised the course of the study.

### Protocol of the study

#### Intraoperative management

The protocol of perioperative management was identical for all patients. The first dose of oral gabapentin 15 mg/kg (max. 600 mg) (Gabapentin TEVA, Teva Pharmaceuticals, Warsaw, Poland) was given 4 h before surgery. For induction to the general anesthesia: fentanyl 1 μg/kg (Fentanyl, Polfa, Poland), propofol 2 mg/kg (Plofed, Polfa S.A, Warsaw, Poland) and rocuronium 0.6 mg/kg (Roqurum, Jelfa SA, Jelenia Góra, Poland) were used. Anesthesia was maintained with a mixture of sevoflurane, oxygen and air (Sevorane, AbbVie, Warsaw, Poland). Thirty minutes before skin incision, acetaminophen 15 mg/kg (Paracetamol Kabi, Fresenius Kabi, Warsaw, Poland), dexamethasone 0.1 mg/kg (Dexaven, SUN-FARM, Łomianki, Poland) and a BF (lidocaine 1.5 mg/kg/30 min or placebo) were applied. During surgery, analgesia was provided with fractionated doses of fentanyl. After induction and before the end of anesthesia, the first two doses of 0.1 mg/kg morphine were administered intravenously (Morphini Sulfas WZF, Polfa S.A, Warsaw, Poland). Intravenous BF infusion (lidocaine 1 mg/kg/h or placebo) was continued during the entire procedure and for 6 h postoperatively.

Intraoperatively, the patient's general condition was monitored by invasive measurement of blood pressure, continuous electrocardiography, pulse oximetry, diuresis measurement, body temperature and assessment of blood biochemical parameters such as acid–base balance, electrolyte and hematocrit levels, glucose and lactates concentrations. The level of anesthesia was monitored by a BIS monitor. The ventilatory frequency was adjusted to obtain an end-tidal carbon dioxide concentration between 35 and 40 mmHg. The neuromuscular blockade was reversed by sugammadex 2 mg/kg (Bridion, Hoddesdon, UK) if necessary.

The patient was extubated on the operating table. After confirming the preserved motor function of the lower limbs, the children were transported to the intensive care unit (ICU), where they remained until the general condition stabilized. Further therapy was continued in the orthopedic ward.

#### Postoperative management

During the preoperative visit, the patients were instructed on the use of the 11-point numerical scale NRS/Numerical Rating Scale/(0 = no pain, 10 = worst imaginable pain) for pain assessment and the use of the PCA device. A protocol for instruction on use the PCA device and the NRS was provided. Two researchers who were responsible for data collection during the study were also properly trained. The severity of pain felt by the child was assessed immediately after surgery and at 2, 6, 9, 15, 24, 30, 40, 48 h postoperatively. In the same time intervals, the intensity of postoperative nausea (the NRS scale: 0 = no nausea, 10 = the worst imaginable form of nausea) and the required antiemetic and analgesic agents were evaluated. PONV (the postoperative nausea and vomiting) was treated with ondansetron 0.1 mg/kg i.v. per dose (Ondansetron Kabi, Fresenius Kabi, Warsaw, Poland) if the NRS for nausea exceeded 3. The time at the oral feeding initiation was recorded, similarly as the first attempt at assuming erect position after surgery. All complications of used therapy were recorded in the patient’s documentation.

Intravenous morphine and non-opioid analgesics were used to treat postoperative pain. For two days after surgery, pain was treated as patient-controlled analgesia (PCA) with i.v. morphine sulfate at a concentration of 1 mg/ml, with a bolus of 1 mg, a lockout-interval—15 min and a maximum dose—0.3 mg/kg/4 h. During the first 16 h after surgery (nighttime), a background infusion of morphine was about 20 μg/kg/h (0.5 mg/h—patients weighing less than 40 kg or 1 mg/h—patients weighing more than 40 kg). With pain reported above 3 NRS, an additional morphine bolus was administered by a blinded non-study nurse (1 mg/kg). After two days, morphine was administered as a single bolus of 0.1 mg/kg via subcutaneous venflon (24G, 0.7 × 19 mm) depending on demand. Postoperatively, morphine consumption was recorded once a day and converted to mg/kg/24 h. The first 24-h morphine measurement included the amount given in the operating room during the operation and that provided by PCA. The first doses of non-opioid analgesics were already administered intravenously during surgery: metamizole 0.5–1 g every 8 h (Pyralgin, Polpharma S.A, Starogard Gdański, Poland) and acetaminophen 15 mg/kg every 6 h. Metamizole dosage was in accordance with the rules in force for over 40 years at the University Pediatric Hospital in Cracow: children under 40 kg–250 mg/10 kg every 8 h, children over 40 kg–1 g every 8 h, the maximum dose of 5 g/day. Oral gabapentin 5 mg/kg (max. 300 mg per dose) was applied every 8 h for three consecutive days.

Patient’s quality of life was evaluated by parents with the Acute Short-form (SF-12) health survey, an abbreviated version of the SF-36. It consists of 12 items, which measures mental and physical components summaries [18]. The survey was conducted in a direct conversation before the operation and by phone 2 months and 4 years after surgery. In addition, one year after the operation, the parents answered by phone the question 5 from SF-12 scale.

### Study outcomes

The primary objective of this investigation was the daily morphine requirements during the first 48 h postoperatively.

Secondary outcome measures were as follows: the severity of the postoperative pain at rest and during coughing assessed using the NRS; the time to start the oral feeding: the clear liquid and the solid diet; the incidence of PONV evaluated with the numeric rating scale for nausea and vomiting registration; the first attempts at sitting and walking after the surgical procedure using 6-min sitting and 6-min walking tests; the quality of life evaluated before, 2 months and 4 years after surgery with the Acute Short-form (SF-12) health survey (parents' assessment); the assessment how much did pain interfere with patients normal work 1 year after surgery, asked by the telephone (interview with parents).

### Laboratory analysis

After 30 min of BF infusion (before the skin incision), immediately after operation, 6 h after surgery and the next morning, blood samples were collected from the arterial line. Routine hematology parameters were determined using a hematological analyzer Sysmex XN-1000 (Sysmex Corp., Japan) and routine biochemistry parameters were measured using a Vitros®5600 (Ortho Clinical Diagnostic, Raritan, USA) analyzer. Plasma concentrations of lidocaine were measured four times, using modified sandwich enzyme-linked immunosorbent assay (ELISA) kits supplied by Neogen Corporation (Lexingon, US). The intra-assay and inter-assay coefficient of variation was 5.2% and 6.7%, respectively. Detection range was 0.005–10.0 µg/ml. Assay was performed without knowledge of whether the sample was from a control or a lidocaine group.

### Sample size calculation

Our sample size was calculated from data obtained from the hospital records, which demonstrated that: the prevalence of spinal surgeries during the year was 60–90 and the mean morphine demand in those patients during hospitalization was approximately 2 mg/kg. In ten arbitrarily chosen patients undergoing major spine operations before the start of the study, opioid consumption during the first 24 postoperative hours was documented, with a mean value of 0.6 mg/kg and a standard deviation of 0.2 mg/kg. We assumed that the administration of lidocaine in the perioperative period will lead to a reduction of the mean morphine demand by 30% per day. The calculation showed that the required sample size (using the above-presented hypothesis) would be 19 patients in each group, with alpha set at 5% and a power of 80%. Finally, the number of children in the lidocaine group was 22 and in the control group 19. The calculations were made according to the formula:

$$k = \frac{{n_{2} }}{{n_{1} }} = 1,$$

$$n_{1} = \frac{{\left( {\sigma_{1}^{2} + \frac{{\sigma_{2}^{2} }}{K}} \right)\left( {z_{1 - \alpha /2} + z_{1 - \beta } } \right)^{2} }}{{\Delta^{2} }},$$

$$n_{1} = \frac{{\left( {0.2^{2} + \frac{{0.2^{2} }}{1}} \right)\left( {1.96 + 0.84} \right)^{2} }}{{0.18^{2} }},$$

$$n_{1} = 19,$$

$$n_{2} = K \times n_{1} = 19,$$

where $\Delta = \left| {\mu_{2} - \mu_{1} } \right|$ is the absolute difference between two means. $\sigma_{1} ,\sigma_{2}$ is the variance of mean #1 and #2. $n_{1}$ is the sample size for group #1. $n_{2}$ is the sample size for group #2. $\alpha$ is the probability of type I error (usually 0.05). $\beta$ is the probability of type II error (usually 0.2). Z is the critical Z value for a given $\alpha$ or $\beta$. *K* is the ratio of sample size for group #2 to group #1.

### Statistical analysis

To compare continuous measurements between both groups, the t-Student or t-Welch test (depending on variance equality assessed by the Levene test) for variables with normal distribution or the *U* Mann–Whitney for variables with a distribution other than normal were used. Normality was assessed by the Shapiro–Wilk test. Categorical variables were compared by the Fisher exact test. The longitudinal measurements were compared using a multivariate linear model for repeated measures. The correlation between results was evaluated by the Pearson correlation test or the Spearman rank-order correlation test, with the Bonferroni correction adjusted for the total number of analyses. The significance level for all the analyses was set at *α* = 0.05. All the tests were two tailed. The analyses were performed using the STATISTICA v.13.5 software (StatSoft Inc., Tulsa, OK, USA).

## Results

**Fig. 1:**
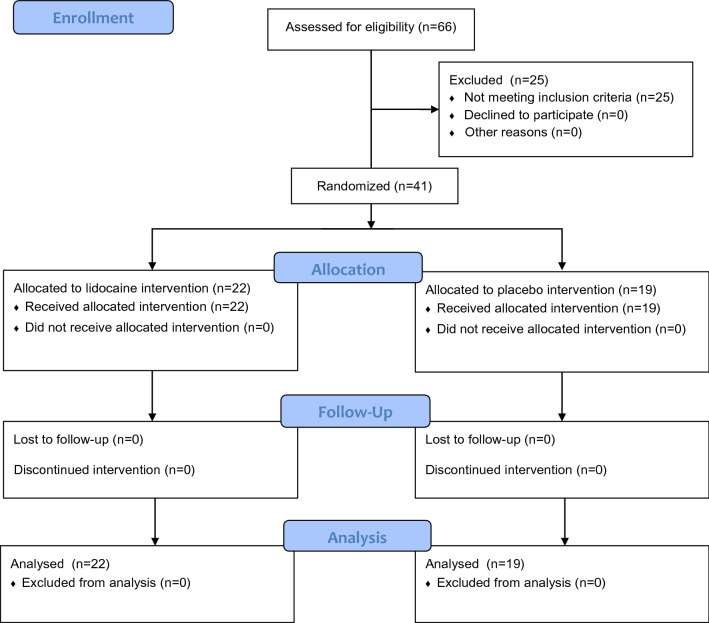
CONSORT trial flow diagram

The study flow chart is shown in Fig. 1. 66 patients planned for elective major spine surgery were assessed for eligibility. 25 children were excluded from the study because they did not meet the inclusion criteria. The remaining forty-one children were randomly assigned to the lidocaine group (number of patients = 22) and to the control group (number of patients = 19). All patients received the allocated treatment. All enrolled patients completed the study and were analyzed. There were no differences in the remaining variables characterizing patients, surgical procedures and the general anesthesia course in both groups (Tables 1, 2).

**Table 1:** Summary of baseline data for the randomized groups

Variable	Lidocaine *n* = 22	Control *n* = 19	*p* value
Age, years	13 (8–15)	13 (9–15)	0.986
BMI, percentile	48 (9–93)	41.5 (9–90)	0.878
Males, *n* (%)	9 (40.9)	8 (42.1)	0.938
ASA, *n* (%)			
I	10 (45.5)	10 (52.6)	0.645
II	12 (54.5)	9 (47.4)	0.645

Categorical variables were presented as counts and percentages; continuous variables were expressed as a median and interquartile range (Q1–Q3)

*BMI* body mass index, *ASA* American Society of Anesthesiologists Physical Status, *n* number of patients

**Table 2:** Summary of intra- and postoperative data for the randomized groups

Variable	Lidocaine *n* = 22		Control *n* = 19		Difference between groups (95% CI)	*p* value
Intraoperative data						
Superior vertebral region, *n* (%)						
Cervical	1 (4.5)		1 (5.3)		–	0.916
Thoracic	3 (13.6)		2 (10.5)		–	0.922
Thoracolumbar	16 (72.7)		15 (78.9)		–	0.643
Lumbosacral	2 (9.1)		1 (5.3)		–	0.639
Use of instrumentation, *n* (%)	22 (100)		19 (100)		–	0.999
Number of operated levels	13 (9–13)		13 (8–14)		–	0.922
Duration of surgery, min	260 (170–285)		300 (270–340)		37.45 (− 3.22 to 121.68)	0.057
Duration of anesthesia, min	335 (225–355)		368 (225–385)		37.14 (− 7.03 to 132.35)	0.054
Fentanyl, μg/kg/h of anesthesia	1.5 (1.36–2.1)		1.3 (1.16–2.19)		− 0.01 (− 0.39 to 0.36)	0.465
Average BIS	45 (40–48)		49 (47–52)		2.12 (− 1.44 to 5.68)	0.14
Mean MAP, mmHg	60 (58–69)		70 (65–74)		12.45 (− 0.22 to 16.67)	0.054
Average heart rate, bpm	77 (71–86)		85 (74–100)		6.88 (− 2.89 to 15.75)	0.083
Postoperative data						
First oral liquid administration, h	3.5 (3–5.5)		7.25 (6–11)		2.88 (1.68 to 4.13)	**0.024**
First oral feeding (solid food), h	14 (13.5–21)		20.5 (19–26)		5.18 **(**0.91 to 6.42)	**0.012**
Ondansetron usage, mg/kg	0 (0–0.1)		0.08 (0–0.2)		0.09 (− 0.24 to 0.21)	0.426
Ondansetron usage, *n* (%)	9 (40.1)		11 (57.9)		–	0.456
6-min sitting test (6MST), h	54 (46–57)		69 (67–75)		14.14 (5.37 to 20.65)	**0.048**
6-min walking test (6MWT), h	67 (62–71)		82.5 (80–89)		13.55 (7.19 to 29.87)	**0.049**
Side effects (V/TSD/RD), *n*	4/1/2		7/0/1		–	0.894
Discharge home, days	6 (4–9)		8 (7–9)		3.54 (− 2.30 to 9.39)	0.066
Short Form Survey (SF-12)	*n*		*n*			
PCS-12—BS	22	40.7 (± 9.4)	19	44.2 (± 10.6)	3.54 (− 3.75 to 9.20)	0.28
PCS-12—2 month	21	35.1 (± 8.8)	19	38.0 (± 7.6)	2.88 (− 2.39 to 8.16)	0.28
PCS-12—4 year	19	47.5 (± 6.6)	17	43.4 (± 10.3)	− 4.11 (− 0.91 to 1.70)	0.15
MCS-12—BS	22	55.8 (± 5.8)	19	57.1 (± 5.9)	1.32 (− 2.31 to 4.95)	0.47
MCS-12—2 month	21	53.8 (± 10.6)	19	56.2 (± 7.1)	2.36 (− 3.37 to 8.08)	0.41
MCS-12—4 year	19	55.1 (± 5.2)	17	53.4 (± 8.1)	− 1.67 (− 6.13 to 3.03)	0.48
PCS-12 (BS—2 month)	5.6 (± 10.5)		6.2 (± 14.5)		0.57 (− 2.74 to 8.61)	0.89
PCS-12 (BS—4 year)	0.4 (± 12.9)		5.3 (± 16.7)		4.92 (− 3.94 to 17.78)	0.08
MCS-12 (BS—2 month)	2.0 (± 10.9)		0.9 (± 7.4)		− 1.09 (− 7.11 to 4.93)	0.72
MCS-12 (BS—4 year)	8.3 (± 19.5)		9.2 (± 21.3)		0.99 (− 11.93 to 13.92)	0.87

Categorical variables were presented as counts and percentages; continuous variables were expressed as a median and interquartile range (Q1–Q3) or as a mean ± standard deviation

*BMI* body mass index, *ASA* American Society of Anesthesiologists Physical Status, *n* number of patients, *BIS* bispectral index, *MAP* mean arterial pressure, *HR* heart rate, *V/TSD/R* vomiting/transient sensory disturbances/respiratory depression, *SF-12* the Acute Short-form health survey, *PCS-12* Physical Score, *MCS-12* Mental Score, *BS* before surgery, *2 month* 2 months after surgery, *4 year* four years after surgery, *(BS—2 month)* difference between the assessment before and 2 months after surgery, *(BS—4 years)* difference between the assessment before and 4 years after surgery

Bold values indicate the* p* < 0.05

### Primary outcome

The lidocaine group had significantly lower cumulative morphine consumption compared to the control, with a reduction of over 30% at 48 h [0.89 (0.58–1.31) mg/kg vs. 2,730 (1.29 (0.79–1.8) mg/kg, 95% CI 0.46 (0.22–0.52) mg/kg, *p* = 0.0299]. The maximum median daily opioid consumption for both groups was in the first postoperative day, also significantly lower in the lidocaine group [0.58 (0.44–0.7) mg/kg vs. 0.74 (0.65–0.91) mg/kg, 95% CI 0.13 (0.11–0.28) mg/kg, *p* = 0.0122], a reduction of over 20%. Cumulative morphine consumption did not differ significantly between the groups in the second and subsequent postoperative days. The relationship between morphine demand and the children's body weight and sex was analyzed. Significant differences were observed only during the first 24 h after surgery: patients with a body weight of over 40 kg of the lidocaine group had a lower demand for morphine compared to the control group in the same weight range [0.5 (0.5–0.6) mg/kg vs. 0.73 (0.56–0.9) mg/kg, *p* = 0.0122].; in the whole group, female children required more morphine than male children [0.72 (0.58–0.96) mg/kg vs. 0.57 (0.4–0.7) mg/kg, *p* = 0.03].; in the lidocaine group, children weighing less than 40 kg consumed more morphine than children weighing more than 40 kg[0.8 (0.58–1.06) mg/kg vs. 0.5 (0.5–0.6) mg/kg, *p* = 0.019] (Table 3).

**Table 3:** Morphine demand

Morphine demand, differentiation between groups depending on body weight and sex						
Variable	Lidocaine	*n*	Control	*n*	Difference between groups (95% CI)	*p* value
Summary morphine demand during whole hospitalization, mg/kg	1.1 (0.7–1.2)	22	1.7 (1.2–2.4)	19	0.58 (0.19 to 0.78)	**0.048**
Summary morphine demand up to 48 h after surgery, mg/kg	0.89 (0.58–1.31)	22	1.29 (0.79–1.8)	19	0.46 (0.22 to 0.52)	**0.0299**
POD1, mg/kg	0.58 (0.44–0.7)	22	0.74 (0.65–0.91)	19	0.13 (0.11 to 0.28)	**0.0122**
Body weight < 40 kg	0.8 (0.58–1.06)	12	0.63 (0.45–0.95)	8	− 0.21 (− 1.12 to 0.53)	0.53
Body weight > 40 kg	0.5 (0.5–0.6)	10	0.73 (0.56–0.9)	11	0.26 (0.02 to 1.14)	**0.0122**
Male	0.5 (0.44–0.6)	9	0.65 (0.35–0.72)	8	0.16 (− 1.12 to 1.34)	0.67
Female	0.67 (0.56–0.92)	13	0.76 (0.6–1.0)	11	0.02 (− 0.97 to 1.23)	0.86
POD2, mg/kg	0.22 (0.2–0.44)	22	0.4 (0.2–0.7)	19	0.16 (− 0.11 to 0.25)	0.14
Body weight < 40 kg	0.37 (0.18–0.53)	12	0.27 (0.05–0.75)	8	0.06 (− 0.92 to 0.13)	0.67
Body weight > 40 kg	0.25 (0.2–0.29)	10	0.3 (0.16–0.46)	11	0.10 (− 0.04 to 0.99)	0.37
Male	0.2 (0.16–0.29)	9	0.25 (0.05–0.7)	8	0.08 (− 0.74 to 0.11)	0.88
Female	0.3 (0.2–0.44)	13	0.3 (0.18–0.46)	11	0.01 (− 0.57 to 0.04)	0.97
Next days, mg/kg	0.17 (0.0–0.39)	22	0.18 (0.0–0.6)	19	0.007 (− 0.06 to 0.40)	0.52
Body weight < 40 kg	0.25 (0.0–0.37)	12	0.16 (0.0–0.76)	8	0.11 (− 1.23 to 1.12)	0.49
Body weight > 40 kg	0.25 (0.11–0.39)	10	0.18 (0.09–1.0)	11	0.09 (− 0.06 to 1.17)	0.43
Male	0.24 (0.1–0.4)	9	0.13 (0.05–0.73)	8	0.14 (− 0.06 to 1.42)	0.84
Female	0.14 (0.0–0.33)	13	0.21 (0.0–1.0)	11	0.21 (− 0.06 to 1.53)	0.46

Morphine demand, differentiation in groups depending on body weight and sex						
Variable	Body weight		*p* value	Sex		*p* value
Lidocaine						
POD1, mg/kg	< 40 kg	> 40 kg	**0.019**	Male	Female	0.14
POD2, mg/kg	< 40 kg	> 40 kg	0.15	Male	Female	0.22
Next days, mg/kg	< 40 kg	> 40 kg	0.19	Male	Female	0.79
Control						
POD1, mg/kg	< 40 kg	> 40 kg	0.62	Male	Female	0.23
POD2, mg/kg	< 40 kg	> 40 kg	1.0	Male	Female	0.77
Next days, mg/kg	< 40 kg	> 40 kg	0.87	Male	Female	0.84

Morphine demand in the whole study group depending on sex and body weight						
Variable	Female	*n*	Male	*n*	*p* value	
Summary morphine demand during whole hospitalization, mg/kg	1.32 (1.0–2.1)	24	1.01 (0.8–1.3)	17	0.12	
Summary morphine demand up to 48 h after surgery, mg/kg	1.05 (0.77–1.46)	24	0.76 (0.7–0.9)	17	0.6	
POD1, mg/kg	0.72 (0.58–0.96)	24	0.57 (0.4–0.7)	17	**0.03**	
POD2, mg/kg	0.3 (0.2–0.45)	24	0.2 (0.1–0.3)	17	0.25	
Next days, mg/kg	0.19 (0.0–0.53)	24	0.15 (0.1–0.4)	17	0.99	
	< 40 kg	*n*	> 40 kg	*n*	*p* value	
Summary morphine demand during whole hospitalization, mg/kg	1.21 (0.95–2.15)	20	1.15 (0.8–1.48)	21	0.74	
Summary morphine demand up to 48 h after surgery, mg/kg	1.03 (0.73–1.65)	20	0.8 (0.67–1.09)	21	0.13	
POD1, mg/kg	0.68 (0.58–1.06)	20	0.6 (0.5–0.73)	21	0.17	
POD2, mg/kg	0.3 (0.13–0.53)	20	0.21 (0.2–0.3)	21	0.37	
Next days, mg/kg	0.08 (0.0–0.43)	20	0.24 (0.1–0.4)	21	0.2	

Categorical variables were presented as counts; continuous variables were expressed as a median and interquartile range (Q1–Q3)

Bold values indicate the* p* < 0.05

*POD* postoperative day, *n* number of patients

### Secondary outcomes

**Fig. 2:**
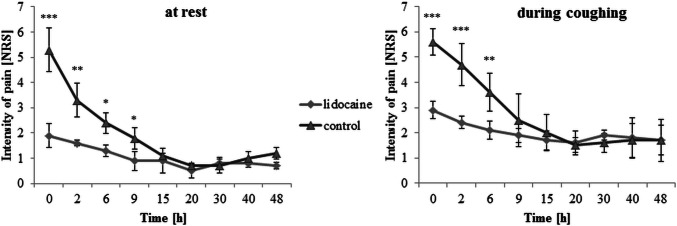
Numerical Rating Scale (NRS) pain score in both groups, at the rest and during coughing, during the first 48 postoperative hours. The significant difference was found for the mean NRS for pain between the two groups (at rest: main effect time: *p* = 0.000005; main effect group: *p* = 0.0062; interaction effect: *p* = 0.041; during coughing: main effect time: *p* = 0.0002; main effect group: *p* = 0.0014; interaction effect: *p* = 0.033). Data are mean ± standard deviations. Asterisks represent statistically significant difference between the groups: *< 0.05, **< 0.01 and ***< 0.005

**Fig. 3:**
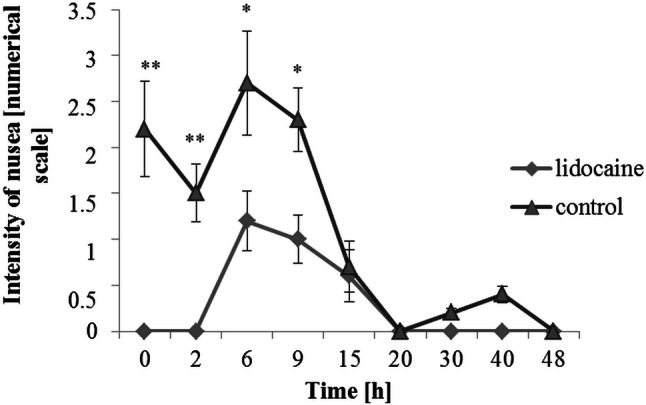
Numerical Rating Scale (NRS) nausea score in the lidocaine and control groups during the first 48 postoperative hours. The significant difference was found for the mean NRS for nausea between the two groups (main effect time: *p* = 0.00035; main effect group: *p* = 0.0029; interaction effect: *p* = 0.0027). Data are mean ± standard deviations. Asterisks represent statistically significant difference between the groups: *< 0.05 and **< 0.01

The cumulative morphine consumption for entire postoperative period was lower in the lidocaine group compared to control, with a reduction of approximately 35% [1.1 (0.7–1.2) vs. 1.7 mg/kg, 95% CI 0.58 (0.19–0.78) mg/kg, *p* = 0.04] (Table 3). Patients from the lidocaine group had lower severity of pain at the rest up to 9 h postoperatively, and during coughing up to 6 h. The difference between the groups in pain sensation at rest, assessed by the NRS at individual time points was: immediately after surgery 1.86 (± 0.46) vs. 5.28 (± 0.85), *p* = 0.00012; 2 h after surgery 1.62 (± 0.12) vs. 3.3 (± 0.67), *p* = 0.00995; 6 h after surgery 1.3 (± 0.22) vs. 2.4 (± 0.41), *p* = 0.0397; 9 h after surgery 0.9 (± 0.37) vs. 1.8 (± 0.42), *p* = 0.049. The difference between the groups in pain sensation during coughing, assessed by the NRS at individual time points was: immediately after surgery 2.86 (± 0.35) vs. 5.5 (± 0.53), *p* = 0.0051; 2 h after surgery 2.43 (± 0.25) vs. 4.78 (± 0.84), *p* = 0.0037; 6 h after surgery 2.1 (± 0.35) vs. 3.56 (± 0.75), *p* = 0.038. In subsequent time measurement points, pain severity was maintained at the comparable level in both groups (Fig. 2). The severity of nausea was higher up to 9 h postoperatively in the control group. The difference between the groups in the severity of nausea assessed by the NRS at individual time points was: immediately after surgery 0 (± 0.0) vs. 2.2 (± 0.52), *p* = 0.0012; 2 h after surgery 0 (± 0) vs. 1.5 (± 0.3), *p* = 0.006; 6 h after surgery 1.2 (± 0.32) vs. 2.7 (± 0.56), *p* = 0.014; 9 h after surgery 1 (± 0.26) vs. 2.3 (± 0.34), *p* = 0.031 (Fig. 3). Both groups did not differ with respect to the postoperative administration of i.v. ondansetron and the incidence of vomiting (Table 2). In the lidocaine group, the liquid diet [3.5 (3–5.5) h vs*.* 7.25 (6–11) h, 95% CI 2.88 (1.68–4.13) h, *p* = 0.024] and the solid food [14 (13.5–21) h vs. 20.5 (19–26), 95% CI 5.18 (0.91–6.42) h, *p* = 0.012] were introduced sooner (Table 2). The patients anesthetized with lidocaine were faster to assume the erect position [sitting 54 (46–57) h vs. 69 (67–75) h, 95% CI 14.14 (5.37–20.65) h, *p* = 0.048; walking: 67 (62–71) h vs. 82.5 (80–89) h, 95% CI 13.55 (7.19–29.87) h, *p* = 0.049] (Table 2).

Plasma lidocaine concentrations 30 min after the start of the infusion were 0.549857 (± 0.2079). The highest drug concentration was recorded immediately after surgery 2.821333 (± 0.65) (Table 4). The number of surgery-associated complications were comparable in both groups (Table 2). No signs of local anesthetic systemic toxicity or serious neurological and cardiac disturbances, like dizziness, convulsions, prolonged/refractory hypotension or arrhythmias, respiratory and cardiac arrest were noted in any of patients receiving lidocaine. One patient at the application site had transient sensory disturbances.

**Table 4:** Plasma lidocaine concentrations

Time	Lidocaine concentration
After 30 min of lidocaine infusion, before skin incision	0.549857 (± 0.2079)
Immediately after surgery	2.821333 (± 0.65)
Six hours after surgery	2.579143 (± 0.7)
Next morning	0.084952 (± 0.08)

Continuous variables were expressed as a mean ± standard deviation

There was no statistical difference in hospital stay after surgery between both groups (Table 2). Two months after the operation, no telephone connection was obtained with legal guardians of one patient from the lidocaine group; after a year with two patients from the lidocaine group; after 4 years with three patients from the lidocaine group and two patients from the control group.

The SF-12 generic health survey did not differ between groups, both in terms of physical and mental health concerns before operation, 2 months and 4 years after surgery (Table 2). Preoperative pain moderately or more disturbing normal activities occurred in 17/41 (41.5%) children surveyed. Two months after surgery in 18/40 (45%) patients. Chronic pain affecting the normal activity of children in the whole group to a moderate and greater degree occurred after a year in 14/38 children and after 4 years in 13/36 children. This represents, respectively, 36.8% and 36.1% of the entire study group. There was no significant statistical difference in the occurrence of preoperative and chronic postoperative pain between the lidocaine and control groups (Table 5).

**Table 5:** Answers to question five from Acute Short-form (SF-12) health survey: during the past 4 weeks, how much did pain interfere with your normal work (including both work outside the home and housework)?

Variable	Lidocaine	Control	*p* value
**Before surgery, *n* (%)**	*n* = 22	*n* = 19	
Not at all	7 (31.8)	9 (47.4)	
A little bit	5 (22.7)	3 (15.8)	0.87
Moderately	8(36.4)	5 (26.3)	
Quite a bit	1 (4.5)	1 (5.3)	
Extremely	1 (4.5)	1 (5.3)	
**Two months after surgery,*n* (%)**	*n* = 21	*n* = 19	
Not at all	4 (19.04)	4 (21.05)	
A little bit	4 (19.04)	10 (52.6)	
Moderately	10 (47.6)	4 (21.05)	0.11
Quite a bit	3 (14.3)	1 (5.3)	
Extremely	0 (0)	0 (0)	
**One year after surgery,*n* (%)**	*n* = 19	*n* = 19	
Not at all	8 (42.1)	3 (15.8)	
A little bit	3 (15.8)	10 (52.6)	
Moderately	7 (36.8)	4 (21.1)	0.56
Quite a bit	1 (5.3)	2 (10.5)	
Extremely	0 (0)	0 (0)	
**Four years after surgery,* n* (%)**	*n* = 19	*n* = 17	
Not at all	7 (36.85)	4 (23.5)	
A little bit	7 (36.85)	5 (29.4)	
Moderately	5 (26.3)	7 (41.2)	0.53
Quite a bit	0 (0)	1 (5.9)	
Extremely	0 (0)	0 (0)	

Categorical variables were presented as counts and percentages

*n* number of patient

## Discussion

The most important finding of the current study was that the perioperative intravenous infusion of lidocaine reduced by 30% the morphine requirements during the first 48 h after major spine surgery in children. Especially on the first day after surgery, patients in the study group consumed less opioids than those in the control group. We also found that in the first 24 h after surgery in the lidocaine group, patients with higher body weight required less morphine than patients with lower body weight. We noted a lower intensity of the postoperative pain in patients receiving lidocaine, especially for up to 6 h after surgery, when the blood serum lidocaine concentration was maintained between 2 and 3 ug/ml. We observed, that in the entire postoperative period children, who received lidocaine, as a part of the applied analgesic therapy regimen, had a lower demand for morphine by approximately 35%. We also found a relationship between sex and the opioids demand in the postoperative period: female patients needed more morphine in the first day after surgery then male patients.

In our study, we followed the principles of preventive multimodal analgesia by initiating therapy with analgesics and coanalgetics with diversified mechanisms of action prior to noxious stimulus appearance, and continuing the treatment during and after surgery [19]. Such a procedure, ensuring multidirectional protection of central nervous system from the intraoperative increased afferent nociceptive stimulation and reduction of peripheral and central sensitization, was aimed to improve the postoperative analgesia and prevent the development of chronic postoperative pain [4]. The analgesic effect of lidocaine is diversified. This drug has peripheral and central actions, which reduces neural responses to pain. Animal studies show that systemic lidocaine alters conduction in neurons of the dorsal horn, dorsal root ganglion and hyperexcitable neuromas without producing nerve conduction block [20]. In vitro, a low dose of lidocaine inhibits voltage-gated sodium channels (VGSC), the glycinergic system, some potassium channels and G-protein coupled receptors, while the higher—voltage-gated calcium channels, NMDA receptors and other potassium channels [21]. After damage to peripheral nerves, there is a high expression of sodium channels on their cell membranes which causes persistent spontaneous discharges that maintain the central nervous system in a state of hyperactive [22]. Lidocaine probably by the inhibition of Na channels (all isoforms) as well as NMDA and G-protein-coupled receptors suppresses spontaneous impulses generated from injured nerve fibers and the proximal dorsal root ganglion [23].

Lidocaine also reduces neurogenic inflammation at the site of tissue injury, by inhibiting granulocyte migration, and reducing the release of lysosomal enzymes and thus pro-inflammatory and anti-inflammatory cytokines [24]. This effect probably is achieved by inhibition of VGSC, G-protein coupled receptors and ATP-sensitive potassium channels. This systemically administered local anesthetic also has a desensitizing effect on TRP (transient receptor potential) channels, which are key components in nocioception and neurogenic inflammation, what explains its long-lasting analgesic effect [21, 25]. Lidocaine limits peripheral and central sensitization. This antihyperalgesic effect is mainly the result of suppression of the neuro-inflammatory response to pain, blockade of neural transmission, inhibition of NMDA receptor and modulation of the glycinergic system [21, 26, 27]. Low-dose lidocaine improves glycinergic signaling, and high dose inhibits it. Its metabolites: *N*-ethylglycine (NEG) is a substrate of the glycine reuptake transporter, and monoethylglycinexylidide (MEGX) inhibits the glycine transporter [27]. Recent studies report that perioperative infusion of lidocaine may also have an analgesic effect by affecting postoperative serum concentrations of endocannabinoids, *N*-acylethanolamines (NAE) and endogenous opioids: β-endorphin, enkephalin and dynorphin [28, 29].

Numerous studies and meta-analyzes have shown a positive effect of the perioperative systemic lidocaine administration in adults on the postoperative analgesia, gastro-intestinal recovery and duration of hospitalization in major surgery [9, 10, 13, 17, 21, 30]. However recent meta-analyzes suggest that lidocaine exerts a positive analgesic effect only in abdominal surgery [11, 12]. Benefits of lidocaine usage in spine surgery in adults have not been thoroughly investigated yet [3, 16, 31, 32]. The results of the few studies conducted in the adults population are ambiguous: Farag et al. reported that i.v. lidocaine significantly improved the postoperative analgesia, and patients undergoing complex spine surgery receiving lidocaine had significantly higher physical SF-12 scores, evaluated one month and three months after surgery than from the placebo group; there were no differences between the groups in PONV intensity and time of hospitalization [3]; Kim et al. found that i.v. lidocaine decreased the severity of the postoperative pain, the consumption of opioids and reduced the length of hospital stay after microdiscectomy surgery [32]; Ibrahim et al. reported that lidocaine significantly reduced hospitalization time and the postoperative pain for up to three months after spine fusion surgery [16]. Dewiter et al. observed no effect of lidocaine on the postoperative pain severity, morphine requirements, PONV, the intensity of the postoperative inflammatory reaction, the hospitalization time and quality of life evaluated one month postoperatively. In the subgroup of patients aged 12–18 years undergoing scoliosis correction, they did not notice significant difference in the mean opioid consumption within the first 24 h after surgery between the lidocaine and the placebo groups [31].

Our study is one of the first to determine the use of intravenous continuous lidocaine infusion in the perioperative period in children. The strength of this study was the small heterogeneity of the group: all our patients were of a similar age, without chronic analgesic treatment before surgery, not burdened with serious diseases causing organ failure, underwent extensive surgery with instrumentation of several levels of the spine. Our type of anesthesia differed from that of the other spine trials. In the anesthesia protocol, according to the principle of multimodal analgesic therapy, in addition to fentanyl and morphine, we used several analgesics and co-analgesics like acetaminophen, metamizole, dexamethasone and gabapentin. Each of these non-opioid pain medications and adjuvants spontaneously reduces the need for morphine. These drugs were a fixed point in the anesthesia protocol in both the study and control groups; therefore, their own effect on the morphine demand did not distinguish between both groups.

Our results showed that systemic lidocaine improved the postoperative gastrointestinal function. The average time of the first intake of liquid diet and solid food in children from the lidocaine group was significantly shorter in comparison with the control group. Patients from the lidocaine group also showed less severe postoperative nausea, especially during the first 9 h after surgery, and the frequency of vomiting and the supply of antiemetics was lower, although without statistical difference. As it is known, the transient postoperative gastrointestinal obstruction is caused by: enteritis-evoking cytokines, that are released to the circulating blood in consequence of surgical trauma; the activation of the sympathetic system and the opioid therapy [1, 29]. Lidocaine, most likely through decreased opioid requirements, anti-inflammatory properties and direct inhibition of sympathetic celiac plexus, accelerates restoring normal gastrointestinal function [14, 17, 30].

The proper treatment of pain, limited opioid consumption and less intensity of PONV probably increases the postoperative comfort experienced by patients, which allows for their effective early rehabilitation. We found that the functional walking capacity as measured by a 6MWT (6-min walking test) distance increased significantly in lidocaine-treated children. Finally, we observed that lidocaine reduced the time of hospitalization by an average of 2 days. This result, however, is statistically insignificant, which may be an effect of low numbers of subjects in the groups. Our observations are in line with a Cochrane review made by Kranke et al. confirming the benefits of administration of systemic lidocaine on recovery of bowel function allowing for earlier rehabilitation and shortening the time of hospitalization [17].

Another strong point of our study was determining the quality of life in groups 2 months and 4 years after surgery, and comparing them with baseline values before surgery. To date, there are no studies investigating the perioperative intravenous lidocaine administration, in which the patient's quality of life assessment was performed at such long time after surgery and compared to preoperative values. However, we did not find any differences between the groups, both in terms of physical and mental health concerns at the above-mentioned time points, also with respect to baseline data. Analyzing the fifth point of the SF-12 scale, we assessed the extent to which pain affected children's daily activities. We noticed, that chronic pain to a moderate or higher degree, affected normal activity in more than 35% of children 1 and 4 years after surgery, but we also did not find a significant statistical difference between the studied groups.

The strength of our study was to determine the concentration of lidocaine in the blood during its administration. The optimal plasma concentration of lidocaine and the duration of the infusion required to obtain the best analgesic effect are still unknown. Different receptors and channels are modulated at different plasma lidocaine levels; therefore, it is unclear whether the blood level of lidocaine correlates with analgesic effects in a dose-dependent manner [21]. So far, no studies have been carried out yet to determine the optimal dose, infusion time and lidocaine plasma levels in children which guarantee the best clinical effect. Using the dose and duration of lidocaine treatment described to be effective in other clinical trials, we obtained serum lidocaine concentrations between 2 and 3 ug/ml during its infusion. No signs of local anesthetic systemic toxicity or serious neurological and cardiac disturbances were noted in any of the patients receiving lidocaine.

### Limitations

Our study has many limitations. First, after surgery, morphine consumption was recorded once a day and converted to mg/kg/24 h. Both, the time of application of the first rescue dose after surgery and the total daily number of rescue doses, were not recorded. Second, it should be noted that the aim of our study was to assess the effect of lidocaine, as an element of multimodal therapy, on opioid requirements during the first 48 h postoperative in pediatric patients undergoing major spine surgery. It has not been studied how lidocaine can reduce the morphine demand: whether through its own activity or through synergistic action with other painkillers administered in a multimodal therapy regimen. Third, the questionnaire assessing the quality of life of patients was not completed by children, only by parents. The parental pain catastrophizing and anxiety sensitivity were not evaluated in any validated test, and could have a significant impact on the assessment of the child's state of health and experiences of pain. In addition, we did not receive a response from all respondents 2 months, 1 and 4 years after the operation due to a lack of telephone communication. Fourth, the study was based only on the primary endpoint. It is possible that increasing the number of patients examined will affect the secondary outcomes. Fifth, the study was carried out in one research center. A small number of patients in each group may lead to underestimation of the possible association between the variables (type II error). Designing a multi-center, prospective, randomized trial, with increased sample size and improved statistical power is necessary to overcome these and other possible similar limitations.

## Conclusions

Introducing the principles of preventive multimodal analgesic therapy is necessary to ensure appropriate comfort of the patient both immediately and late after surgery. Achieving this is extremely difficult in children subjected to major surgical procedures where regional anesthesia techniques are controversial. Perioperative lidocaine administration seems to reduce the morphine demand in the postoperative period and accelerate convalescence of pediatric patients after major spine surgery.
